# Fattening in Saline and Alkaline Water Improves the Color, Nutritional and Taste Quality of Adult Chinese Mitten Crab *Eriocheir sinensis*

**DOI:** 10.3390/foods11172573

**Published:** 2022-08-25

**Authors:** Shihui Wang, Kun Guo, Liang Luo, Rui Zhang, Wei Xu, Yingying Song, Zhigang Zhao

**Affiliations:** 1 Key Open Laboratory of Cold Water Fish Germplasm Resources and Breeding of Heilongjiang Province, Heilongjiang River Fisheries Research Institute, Chinese Academy of Fishery Sciences, Harbin 150070, China; 2 Engineering Technology Research Center of Saline-Alkaline Water Fisheries (Harbin), Chinese Academy of Fishery Sciences, Harbin 150070, China; 3 College of Animal Science and Technology, Northeast Agricultural University, Harbin 150030, China

**Keywords:** *Eriocheir sinensis*, aquaculture, fatty acid, free amino acid

## Abstract

The majority of pond-reared Chinese mitten crab (*Eriocheir sinensis*) grow and fatten in freshwater. Previous studies illustrated that *E. sinensis* cultured in saline-alkaline water in outdoor environments showed a higher quality than that cultured in freshwater. However, it is still unclear whether salinity or alkalinity has an important positive effect on the quality of *E. sinensis*. This study aimed to investigate the gonadal development, edible yield, coloration, and nutritional and flavor quality of *E. sinensis* fattening in saline and alkaline water indoors. Results showed that there were no significant changes observed in gonadosomatic index (GSI) and other edible parameters among freshwater (FW), saline water (SW), and alkaline water (AW) during the 55-day fattening period (*p* > 0.05). Significantly higher a* and b* values of freeze-dried female carapace were observed fattening in SW and AW compared with that of FW (*p* < 0.05). The crude protein in gonad and male muscle, moisture in female muscle, and crude lipid in male muscle increased significantly from FW to SW and AW (*p* < 0.05). Better nutritional and flavor values were also detected in male hepatopancreas and muscles. In conclusion, numerous advantages of fattening in SW and AW were observed, including the improvement of carotenoid accumulation in freeze-dried carapace, DHA, EPA, total essential free amino acids (∑EFAA), total free amino acids (∑FAA), and total umami values (∑TUV) contents in male hepatopancreas and muscle.

## 1. Introduction

The Chinese mitten crab *Eriocheir sinensis*, an important freshwater product, has high economic value and is favored by consumers because of its delicious taste and high polyunsaturated fatty acid (PUFA) and amino acid (AA) contents in China [1]. The aquaculture yield reached 808,274 t in 2021, and the output value has increased to beyond CNY 50 billion [2]. *E. sinensis* has a unique life cycle. Juvenile and adult individuals live in freshwater, while mature or nearly mature individuals migrate to the estuary for reproduction [3]. The culture modes of *E. sinensis* mainly include pond culture, lake, or reed pond proliferation, and rice crab co-culture, which are distributed in different regions of China. With increasing pressure for environmental protection, expanding the aquaculture space of aquatic products has become one of the most important elements affecting the development of the *E. sinensis* industry. The area of *E. sinensis* cultured in saline-alkaline water has gradually increased in recent years [4,5].

Globally, saline-alkaline land covers 0.95 billion hectares, accounting for 1/3 of the total land area [6]. The area of saline-alkaline land in China is approximately 99.13 million hectares, and the low-lying saline-alkaline water is approximately 45.87 million hectares, accounting for approximately 55% of the total lake area [7]. Previous studies have demonstrated that *E. sinensis* cultured in saline-alkaline water in outdoor ponds does not affect growth and gonadal development [8]. Meanwhile, *E. sinensis* cultured in saline-alkaline water in outdoor environments show higher levels of long-chain unsaturated fatty acids (LC-PUFA), especially DHA and EPA, than that cultured in freshwater [4,5]. However, the nutritional quality of *E. sinensis* cultured in outdoor earthen ponds can be affected by numerous factors, such as germplasm [9], culture environment [10], and diet [11]. Salinity and alkalinity are important ecological factors in the culture environment [12,13]. Hence, it is still unclear whether salinity or alkalinity has an effect on the nutritional quality of *E. sinensis*, and the main reason why *E. sinensis* accumulates high levels of DHA and EPA in outdoor saline-alkaline earthen ponds.

Since *E. sinensis* is a migratory aquatic animal, salinity plays an important role in its reproduction. Therefore, there are many studies on salinity [14,15,16,17]. The majority of studies have mainly focused on the physiological metabolism and osmoregulation of salinity in *E. sinensis* [14,15]. Only a few studies have paid close attention to the nutritional quality and flavor quality of *E. sinensis* [16,17]. Even though there is little literature, they still focus on brackish water (12 ppt) during the process of reproductive migration or low salinity seawater (7 ppt) for a short time. Previous studies have investigated edible yield and nutritional quality for fattening over 60 days in outdoor ponds [1,11]. Nevertheless, no studies have reported on the gonadal development, color, nutrition, and flavor quality of *E. sinensis* fattening in low salinity water (1.5 ppt) indoors for a long time.

Compared with numerous studies on the salinity of *E. sinensis*, few reports on the alkalinity of *E. sinensis* exist in the literature. More reports have focused on the toxicity of alkalinity on *Tribolodon brandti* [18] and *Macrobrachium nipponense* [19]. A recent study simply illustrated the toxicity of carbonate alkalinity (NaHCO_3_) on *E. sinensis* [20]. However, no future reports have focused on the quality of *E. sinensis* edible tissues.

During the period of fattening, edible parameters, commonly including the hepatosomatic index (HSI), gonadosomatic index (GSI), meat yield (MY), and total edible yield (TEY), are quite important indicators utilized in the evaluation of fattening performance for *E. sinensis* [11]. Meanwhile, color parameter, proximate composition, fatty acid, and free amino acid are also key indicators for the coloration and nutritional and flavor quality of *E. sinensis* [4,21]. Therefore, based on the above reasoning, this experiment was designed to investigate the effects on the gonadal development, edible yield, coloration, and nutritional and flavor quality of *E. sinensis* fattening in saline or alkaline water.

## 2. Materials and Methods

### 2.1. Experimental Design

This experiment was conducted at the Heilongjiang River Fisheries Research Institute, CAFS (Harbin, China). Approximately 140 adult *E. sinensis* post puberty molt (female body weight 80~100 g, male body weight 100~120 g) were obtained from a local crab farm in early August 2021. The crabs were cultured in earth pond outside with *Elodea canadensis* transplanted from 1 May to the sampling time, and fed once a day at 17:00 with a commercial formulated diet (crude protein ≥ 36.0%, crude fat ≥ 5.0%, moisture ≤ 12.0%, ash ≤ 18.0%; Nanjing Aohua Biotechnology Co., Ltd., Nanjing, China). Among them, a total of 120 healthy, active, and intact individuals were selected and conducted for the experiment on 5th August according to our own protocol. The fattening trial was conducted in 12 indoor glass tanks (64 × 38 × 43 cm) with 60 L water in each glass tank. Four glass tanks as one treatment and three treatments were set in this study: freshwater (FW: salinity 0 ppt, alkalinity 0 mmol/L), saline water (SW: salinity 1.5 ppt, alkalinity 0 mmol/L), and alkaline water (AW: alkalinity 10 mmol/L, salinity 0 ppt). Forty individuals together (half females and half males) were randomly distributed, averaging 10 individuals in each glass tank. FW was sourced from tap water. Analytical pure sodium chloride NaCl (Sinopharm Chemical Reagent Co., Shenyang, China) was used to adjust the salinity to 1.5 ppt, and analytical pure sodium bicarbonate NaHCO_3_ (Sinopharm Chemical Reagent Co., Shenyang, China) was used to adjust 10 mmol/L alkalinity. The saline-alkaline concentration in this experiment originated from the outdoor earthen pond from Dongying in Shandong Province and Daqing from Heilongjiang Province of China.

### 2.2. Culture Management

During the fattening period, the water temperature was changed 2 °C every 10 days and presented a downward trend from 25 to 15 °C depending on the season from 5th August to 28th September. In an indoor circulating aquaculture system, around 30% of glass tank water was replaced in each tank every day with dechlorinated tap water at the appropriate temperature and maintained constant water salinity and alkalinity. The ammonia-N, nitrite, salinity, alkalinity, dissolved oxygen (DO), and pH of the water were checked every day. Over the course of the trial, *E. sinensis* was fed daily at 5 pm with a commercial crab diet (crude protein ≥ 36.0%, crude fat ≥ 5.0%, moisture ≤ 12.0%, ash ≤ 18.0%; Nanjing Aohua Feed Co., Ltd., Nanjing, China), and food residue was removed next morning. The feeding amount was adjusted according to Zhang’s study [16].

### 2.3. Sample Collection and Dissection

The studies in *E. sinensis* were reviewed and approved by the Committee for the Welfare and Ethics of Laboratory Animals of the Heilongjiang River Fisheries Research Institute (Approval numbers: 20210915-001, approved on 15 September 2021). On 28 September, a total of 40 individuals from each treatment were sampled. The *E. sinensis* surface was wiped with a towel, and digital balance (JA2002, precision = 0.01 g, Shanghai Puchun Measuring Instrument Co., Ltd., Shanghai, China) was used to measure the body weight of each crab. A Vernier caliper (111-101-10G, precision = 0.01 mm, Guilin Guanglu Measuring Instrument Co., Ltd., Guilin, China) was used to measure the carapace length and width parameters. The *E. sinensis* was then dissected to obtain edible tissues (hepatopancreas, gonad, and muscle). The method and steps of dissection were as follows: First, the carapace and breastplate of *E. sinensis* were separated, and then the hepatopancreas and gonads hidden in the carapace were carefully taken out. Second, the hexagonal heart was discarded, and the remaining hepatopancreas and gonads in the chest were carefully taken out. The edible tissues were weighed for calculating hepatosomatic index (HSI, %), gonadosomatic index (GSI, %), meat yield (MY, %), and total edible yield (TEY, %). Subsequently, all edible tissues were stored separately at −40 °C for further biochemical analysis. The HSI, GSI, MY, TEY, and condition factor (CF, g/cm^3^) were calculated with the following Formulas (1)–(5):

(1) $$\mathrm{HSI}\;(\%)=100\;\times \;\frac{\mathrm{hepatopancreas}\;\mathrm{weight}}{\mathrm{body}\;\mathrm{weight}}$$

(2) $$\mathrm{GSI}\;(\%)=100\;\times \;\frac{\mathrm{gonad}\;\mathrm{weight}}{\mathrm{body}\;\mathrm{weight}}$$

(3) $$\mathrm{MY}\;(\%)=100\;\times \;\frac{\mathrm{meat}\;\mathrm{weight}}{\mathrm{body}\;\mathrm{weight}}$$

(4) $$\mathrm{TEY}\;(\%)=100\;\times \;\frac{\mathrm{hepatopancreas}\;\mathrm{weight}+\mathrm{gonad}\;\mathrm{weight}+\mathrm{meat}\;\mathrm{weight}}{\mathrm{body}\;\mathrm{weight}}$$

(5) $$\mathrm{CF}\;(g/{\mathrm{cm}}^{3})=\frac{\mathrm{body}\;\mathrm{weight}}{{\mathrm{carapace}\;\mathrm{length}}^{3}}$$

### 2.4. Measurements of Color Parameters

A colorimeter (CR-400, Konica Minolta, Marunouchi, Tokyo, Japan) was used to measure the color values (lightness L*, redness a*, and yellowness b*) of freeze-dried carapace, and female gonad (ovary) of *E. sinensis* from three treatments, respectively. Six relatively smooth points on the carapace surface **[21]**, and three random points on the ovary were selected for L*, a*, and b* measures.

### 2.5. Proximate Composition

The moisture analysis of experimental *E. sinensis* edible tissues from the three treatments was determined with a vacuum freeze-dryer (FD-1A-50, Biocoll, Beijing, China) at −50 °C to a constant weight [4]. Prior to the future biochemical analysis, five freeze-dried *E. sinensis* tissues were randomly selected to form a replicate. Three replicates were designed in the experiment. The crude protein, crude lipid, and ash contents were separately determined using the Kjeldahl method [22], GB 5009.6-2016 [23], and AOAC procedures [22], respectively.

### 2.6. Fatty Acid Profile

The peak area percentage method by GB 5009.168-2016 was carried out to measure fatty acids [24]. All samples were repeated three times. Crude lipids extracted from *E. sinensis* edible tissues were further processed for fatty acid analysis. The results are presented as the percentage of each fatty acid with respect to the total fatty acids (%).

### 2.7. Free Amino Acid Analysis and Taste Activity Value

Freeze-dried *E. sinensis* tissue samples were processed according to the previously described method [16]. A total of 17 amino acids were calculated using this method. The taste activity value (TAV) was calculated as the ratio of the concentration of taste compounds measured above in the *E. sinensis* edible tissue to its threshold value [4].

### 2.8. Statistical Analysis

The results are presented as mean values ± standard error (SE). SPSS 22.0 software (SPSS Inc., Chicago, IL, USA) was used for statistical analysis. A one-way ANOVA was used to determine the differences among these three treatments, and Duncan’s multiple range test was carried out for comparisons. The comparison test *p* < 0.05 was regarded as the statistical significance.

## 3. Results

### 3.1. Gonadal Development and Total Edible Yield

**Figure 1:**
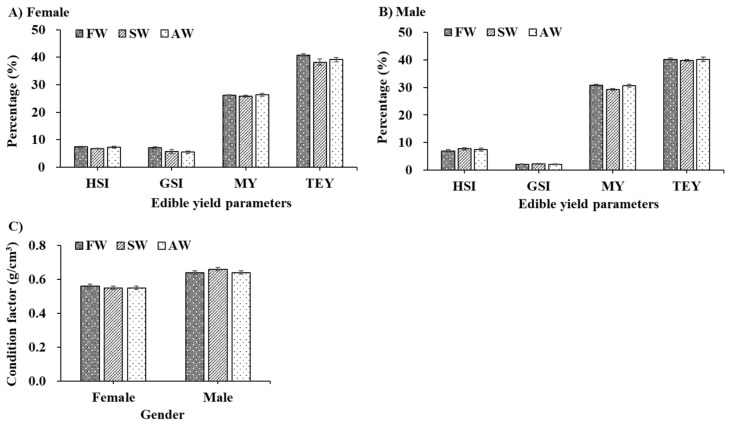
The edible yield (% body weight, (**A**,**B**)) and condition factor (%, (**C**)) of adult *Eriocheir sinensis* from different culture environments. The data are presented as mean ± standard error (SE) (n = 20). FW, freshwater; SW, saline water; AW, Alkaline water; HSI, hepatosomatic index; GSI, gonadosomatic index; MY, meat yield; TEY, total edible yield.

The edible yield and condition factors of adult *E. sinensis* from different culture environments are shown in Figure 1. There was no significantly increasing or decreasing trend observed among the FW, SW, and AW during the 55-day fattening period (*p* > 0.05). However, slight numerical differences were found between edible parameters and the condition factor (CF) among the three treatments at the end of the experiment. For females (Figure 1A,C), the HSI, GSI, TEY, and CF parameters of *E. sinensis* from FW were slightly higher than those of SW and AW. For males (Figure 1B,C), the HSI, GSI, and CF parameters of *E. sinensis* fattening in SW were much better than those of FW and AW, while a higher percentage of *E. sinensis* MY and TEY parameters existed in AW.

### 3.2. Color Parameters

**Figure 2:**
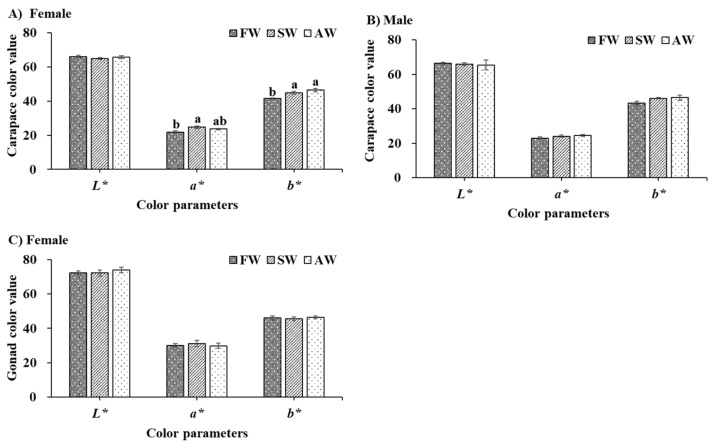
The freeze-dried carapace (**A**,**B**) and gonad (**C**) color quality of adult *Eriocheir sinensis* from different culture environments. The data are presented as mean ± standard error (SE) (n = 20). Different letters indicate a significant difference among different culture environments (*p* < 0.05). FW, freshwater; SW, saline water; AW, Alkaline water; L*, a*, and b* represent the color parameters of lightness, redness, and yellowness, respectively.

Significantly higher a* (redness) and b* (yellowness) values of freeze-dried female carapace were observed fattening in SW and AW compared with that of FW (*p* < 0.05) (Figure 2A). No significant differences existed between the freeze-dried female gonad and male carapace among the three treatments (Figure 2B,C) (*p* > 0.05), but the a* and b* values of freeze-dried male carapace still showed an increasing trend of fattening in SW and AW. Overall, the effects of fattening in SW and AW on the color quality of the carapace are more obvious than that of the female gonad.

### 3.3. Proximate Composition

The proximate composition of adult *E. sinensis* fattening in different culture environments is presented in Table 1. The crude protein in gonad and male muscle, moisture in female muscle, and crude lipid in male muscle increased significantly from FW to SW and AW (*p* < 0.05), whereas a decreasing trend was detected by crude protein in female muscle from FW to SW and AW (*p* < 0.05). The crude lipid of hepatopancreas exhibited an obviously increasing trend of fattening in SW and AW compared with that of fattening in FW, but no significant difference (*p* > 0.05).

### 3.4. Fatty Acid Profiles

The fatty acid composition of adult *E. sinensis* fattening in different culture environments is summarized in Table 2. The fatty acids of *E. sinensis* edible tissues contain saturated fatty acids (SFAs), monounsaturated fatty acids (MUFAs), and polyunsaturated fatty acids (PUFAs).

### 3.5. Free Amino Acid Composition and Taste Activity Value

Concentrations of ∑SFA in male hepatopancreas significantly decreased from FW to SW and AW (*p* < 0.05) (Table 2). This trend was mainly driven by a reduction in 16:0, which decreased from 27.90 ± 0.90% in FW to 23.96 ± 0% in SW. Concentrations of ∑MUFA in male hepatopancreas also decreased from FW to SW and AW, but no significant difference was observed (*p* > 0.05). This trend was generally driven by a reduction in dominant 18:1n9c, which decreased from 46.65 ± 1.61% in FW to 42.76 ± 0.24% in SW. 18:2n6c was present in the highest abundance, followed by 18:3n3, DHA, EPA, and ARA within PUFA. A significantly increasing trend of ∑PUFA in male hepatopancreas was observed from FW to AW and SW (*p* < 0.05), which is generally driven by an increase in the above-mentioned fatty acids. The highest content of DHA in male hepatopancreas was detected in AW compared with that of FW (*p* < 0.05). A significant improvement of ∑HUFA, ∑n-3 PUFA, and ∑DHA + EPA contents was also observed in SW and AW male hepatopancreas (*p* < 0.05).

In the gonad (Table 2), the ovaries and testes had apparent differences in their fatty acid composition and concentration. Females have higher concentrations of ∑SFA, ∑MUFA and ∑EFA, but lower percentages of ∑PUFA, ∑HUFA, ∑n-3 PUFA, ∑n-6 PUFA and ∑DHA + EPA compared with males. A significantly decreasing trend was found in 18:1n9c from FW to SW (*p* < 0.05). The ∑EFA content of the testis in SW and AW was slightly higher than that of FW, but no significant difference was detected (*p* > 0.05).

The lower crude lipid concentration of the testis and muscle was recorded. Therefore, the fatty acid compositions of the muscle were similar to those of the testis, but obviously different from those of the ovary and hepatopancreas (Table 2). Regardless of gender, concentrations of ∑SFA in muscle significantly decreased from FW to SW (*p* < 0.05). This trend was mainly driven by a reduction in 16:0 and 18:0. Whereas the female muscle content of ∑SFA in AW was significantly higher than that of FW, male muscle content was lower compared with that of FW (*p* < 0.05). A significantly decreasing trend was detected of ∑MUFA in the male muscle from FW to SW and AW (*p* < 0.05), which was mainly driven by a reduction in 18:1n9c. The content of ∑PUFA in male muscle significantly increased from FW to SW and AW (*p* < 0.05). This trend was mainly driven by an increase in 18:2n6c, DHA, and EPA. As for the indices describing combinations of PUFA, the contents of ∑HUFA, ∑n-3 PUFA, ∑DHA + EPA showed a significantly increasing trend from FW to SW and AW (*p* < 0.05).

Seventeen FAAs including seven essential free amino acids (EFAAs) for adult human beings, and two tastes including pleasant taste (umami and sweetness) and unpleasant taste (bitterness) are detected, respectively (Table 3 and Table 4).

With respect to total essential free amino acids (∑EFAA) and total free amino acids (∑FAA) of *E. sinensis* hepatopancreas, the concentrations in females significantly increased from FW to SW and AW (*p* < 0.05) (Table 3). This trend was mainly driven by a significant increase in aspartic acid (Asp), cysteine (Cys), histidine (His), proline (Pro), tyrosine (Tyr), isoleucine (Ile), leucine (Leu), lysine (Lys), methionine (Met), phenylalanine (Phe), and threonine (Thr), which ∑EFAA increased from 551.55 ± 18.37 mg/100 g in FW to 696.63 ± 5.77 mg/100 g in SW, and ∑FAA increased from 1476.65 ± 4.09 mg/100 g in FW to 1721.86 ± 46.53 mg/100 g in AW. A similar trend was also observed in males, but no significant difference existed (*p* > 0.05). Regardless of gender, the total umami values (∑TUV) containing Asp and glutamic acid (Glu) in SW and AW were obviously higher than those of FW, which increased from 3.89 to 4.47 in females and from 3.40 to 3.89 in males with the domain umami compound Glu (Table 4). Nevertheless, the total sweetness values (∑TSV) containing alanine (Ala), glycine (Gly), serine (Ser), Thr, and Pro in females and total bitterness values (∑TBV) including arginine (Arg), Lys, valine (Val), Met, histidine (His), Ile, Leu, and Phe in males, were slightly decreased from FW to SW and AW. A slightly increasing trend of ∑TSV in males and ∑TBV in females was detected from FW to SW and AW. The domain sweetness compound was Ala, and the bitterness compounds were Arg, Lys, Val, Met, and His, respectively.

Except for the female lower ∑EFAA in AW, the contents of ∑EFAA increased from FW to SW and AW in gonad (Table 3). Due to the low content of female ∑FAA in FW, the percentage of ∑EFAA to ∑FAA (PETFAA) was higher than that of AW and SW. The concentrations of His, Pro, and Lys in females significantly increased from FW to SW and AW (*p* < 0.05), while a similar trend was also observed in males with higher contents of His, Ile, Leu, and Thr between SW and AW (*p* < 0.05). For the ovary, the ∑TUV and ∑TSV fattening in SW and AW were higher than that of FW (Table 4), indicating an obvious improvement in umami and sweetness. An apparent increase of ∑TBV was also found in SW compared with that of FW. The domain umami compound was Glu, while Ala was the main sweetness compound. Arg, Lys, and His presented as an unpleasant taste. For the testis, the ∑TUV, ∑TSV and ∑TBV fattening in FW were slightly higher than that in SW, but lower than that in AW. The testis possessed similar umami and sweetness compounds but a lower bitterness compound content compared with the ovary.

With respect to ∑EFAA and ∑FAA of the *E. sinensis* muscle, an apparent improvement was detected between SW and AW compared with that of FW (Table 3). This trend was mainly driven by an obvious increase in Gly, and Pro. Significant change was observed among the three treatments including Arg, Gly, Pro, Lys, and ∑FAA in females, as well as His, Pro, and Tyr in males (*p* < 0.05). Regardless of gender, the ∑TUV in SW and AW was obviously higher than that of FW, which increased from 1.31 to 2.08 in females and from 1.83 to 2.53 in males with the domain umami compound Glu (Table 4). A similar increasing trend was also observed for ∑TSV between SW and AW, with the values ranging from 9.48 to 11.28 in females and from 9.21 to 11.33 in males, accompanied by domain sweetness compounds Ala, Gly, and Pro. For ∑TBV, Arg contributed the largest amount of flavor compound with the TAV > 9. 

## 4. Discussion

### 4.1. Total Edible Yield

Fattening is a highly important aspect in the farming of *E. sinensis*, where fattening performance can generally be evaluated by gonadal development status and edible yield [25]. *E. sinensis* with well-developed gonadal systems are usually sold at a higher price than less developed *E. sinensis*; therefore, the status of gonadal development directly affects the nutritional value and price of the market [11]. The present study showed that *E. sinensis* GSI parament fattening in SW and AW was not significantly different from that of fattening in FW (*p* > 0.05), which illustrated that low salinity and alkalinity could not significantly affect the gonadal development of *E. sinensis*. Nevertheless, a downward trend in GSI was detected in females, which is likely caused by the lower body weight of *E. sinensis* in SW and AW. In the same polyculture pond, the smaller the body weight of adult individuals, the earlier the puberty molt is completed. That is, the gonadal development of small body weight starts earlier than that of big body weight. Previous studies have argued that no significant GSI change was detected, fattening below 6 ppt salinity [14], and this result was consistent with our study.

### 4.2. Color Parameters and Biochemical Composition

The market value of *E. sinensis* is predominately driven by its visual appearance. Generally, the reddish color of crustaceans means higher market prices [26]. The color of *E. sinensis* is attributable to the deposition of carotenoids, especially astaxanthin [21]. In the present study, higher a* values of freeze-dried carapace were observed fattening between SW and AW compared with that of FW, illustrating higher astaxanthin and canthaxanthin contents [21,27]. A similar trend was also observed with higher b* values of freeze-dried carapace fattening between SW and AW, suggesting strongly zeaxanthin and β-carotene contents [27]. These results suggest that fattening in saline-alkaline water was helpful to carotenoid accumulation in *E. sinensis* carapace. A similar conclusion was also detected that salinity stress induced an increase in carotenoid content, such as *Synechocystis* [28], and *Golenkinia* [29].

The biochemical composition of edible tissues is an important indicator for evaluating the nutritional value of aquatic animals, and its composition is influenced by many factors, such as germplasm, culture environment, fattening stage, and diet [1,25]. This study showed that male *E. sinensis* fattening in SW and AW had lower moisture contents in muscle and hepatopancreas, but had higher protein contents in muscle. The possible explanation is that salinity or alkalinity affects osmotic pressure regulation, resulting in a decrease in the moisture of *E. sinensis* edible tissues, while the higher protein content may be due to the self-protection strategy adopted to resist the environment. Similar results were also observed in *Scylla paramamosain* [30]. Female fattening in SW and AW had lower crude protein compared with that of FW, implying that *E. sinensis* muscle tissue of different genders was reflected differently under SW and AW.

### 4.3. Fatty Acid Composition

Fatty acid composition is an important indicator for the evaluation of the quality of edible aquatic species, especially essential fatty acids, and unsaturated fatty acid contents [25]. In this study, the concentrations of ∑SFA significantly decreased, but the content of ∑PUFA significantly increased in male hepatopancreas from FW to SW and AW (*p* < 0.05). This result was consistent with previous studies [15,17], which may explain why it is necessary to improve membrane permeability to enhance the absorption of ions and maintain the intracellular ionic balance. Hence, increasing ∑PUFA levels might be beneficial to the intracellular and extracellular osmotic and ionic balance [15].

Balanced concentrations of fatty acids are essential for human health, which mainly refer to a higher proportion of essential fatty acids (EFA) and LC-PUFAs [31]. In this study, except for slightly low ∑EFA in SW female muscle, the ∑EFA contents of *E. sinensis* other edible tissues fattening in SW were higher than those of fattening in FW, illustrating a higher quality. DHA, EPA, and ARA are three important LC-PUFAs for human health, especially fetuses, infants, adolescents, and pregnant or lactating women [32]. DHA and EPA can inhibit the proliferation of tumor cells [33]. This indoor experiment demonstrated that fattening in SW and AW could improve the DHA and EPA contents in male hepatopancreas and muscle, which illustrated that salinity and alkalinity could regulate the accumulation of DHA and EPA and promote endogenous biosynthesis. The internal mechanism may be to increase the protein expression of the elongase of very long-chain fatty acid (Elovl) and fatty acid desaturase (Fad) in the process of LC-PUFA synthesis [34,35]. However, through the comparison between indoor and outdoor earthen pond cultures [4], our results support that the *E. sinensis* LC-PUFA accumulation mainly comes from exogenous food sources, followed by endogenous biosynthesis.

Long-chain n-3 and n-6 PUFAs and their ratios (n-3/n-6) are also considered to be significantly important for human health. The FAO/WHO [31] recommended that the appropriate dietary n-3/n-6 PUFA ratio was 0.1~0.2. If the ratio was > 0.2, it would be better for human health [1]. The results in this study illustrated n-3/n-6 PUFA ratios of all the *E. sinensis* edible tissues were > 0.1, suggesting that fattening in SW and AW would not affect the nutritional quality. However, compared with previous studies [1,4,11], a lower n-3/n-6 PUFA ratio in hepatopancreas was observed in this study. This is likely attributable to the black color in hepatopancreas of *E. sinensis* rearing in an indoor circulating aquaculture system because above 40% fatty acid parameters were significantly changed and a lower n-3/n-6 PUFA ratio was found between normal color and black color in *E. sinensis* hepatopancreas [36].

### 4.4. FAA Composition and TAV Analysis

It is widely known that FAA composition and concentration play a marked role in nutritional and non-volatile flavor quality (taste) [5,25]. Generally, Asp and Glu contribute to umami, and Ser, Gly, Thr, Pro, and Ala contribute to sweetness. His, Phe, Ile, and Leu contribute to bitterness [4,16,25], while the taste of Arg relies on its concentration. Regardless of gender, the present study showed that the ∑EFAA and ∑FAA contents of *E. sinensis* edible tissues in hepatopancreas and muscle increased fattening in SW and AW, which explained the higher nutritional quality. Similar results were also detected by previous findings [17,37].

TAV is generally used as the most classical and objective method to determine the taste intensity of a single compound in food and to evaluate its contribution to overall flavor quality [37]. Compounds with a TAV > 1 were considered to significantly contribute toward *E. sinensis* taste, while compounds with a TAV < 1 were considered to contribute less [16]. This study showed that the compositions of main flavor amino acids are consistent regardless of fattening in FW or saline-alkaline water, but the contents of the main flavor amino acids are different. Similar changes have been confirmed by previous studies [16,17]. Glu, Ala, and Arg are likely the main compounds for the strong umami and sweet taste of *E. sinensis* edible tissues. This result is similar to previous studies [4,5,16,17,25]. Although Arg has a bitter taste, abundant Arg can enhance the persistence, complexity, and strong sense of umami [38]. Even in Wang and Zhang’s research, Arg was directly listed as a pleasant taste amino acid [16,37]. Studies have confirmed Arg’s great contribution to the overall taste of aquatic products, and showed a positive correlation [16,39]. Previous studies have argued that Pro, Ala, Gly, and Arg may be used as osmotic regulators for crustacean exposed to salinity for extensive time periods [14], and this may be an important reason for the above FAA changes in the process of fattening in saline-alkaline water. Further, some obvious changes in FAAs were also detected in fattening between SW and AW. Although Na^+^ was the common cation, different anions (salinity, Cl^−^; alkalinity, HCO_3_^−^) may have led to the above differences. However, how salinity and alkalinity regulate the difference in FAA content still needs further research.

## 5. Conclusions

In the present study, no significantly increasing or decreasing trend was observed in GSI or TEY among FW, SW, and AW during the 55-day fattening period. Higher a* and b* values of freeze-dried carapace were observed in the fattening between SW and AW. The crude protein in gonad and male muscle, moisture in female muscle, and crude lipid in male muscle increased significantly from FW to SW and AW. Better nutritional and flavor values were also detected in male hepatopancreas and muscle. In summary, numerous advantages of fattening in SW and AW were observed, including the improvement of carotenoid accumulation in freeze-dried carapace, DHA, EPA, ∑EFAA, ∑FAA, and ∑TUV contents in male hepatopancreas and muscle. These results will be helpful in improving the quality of *E. sinensis*. However, the synthetic mechanism inside organisms needs to be further studied.

## Figures and Tables

**Table 1:** The proximate composition (% wet weight) of adult *Eriocheir sinensis* fattening in different culture environments.

Item	Female			Male		
	FW	SW	AW	FW	SW	AW
**Hepatopancreas**						
moisture	59.50 ± 1.60	59.62 ± 3.39	61.04 ± 2.02	55.29 ± 0.66	50.34 ± 4.16	51.48 ± 3.26
crude protein	10.86 ± 0.51	9.06 ± 0.07	9.94 ± 0.86	8.96 ± 0.11	9.28 ± 2.75	8.01 ± 0.85
crude lipid	24.10 ± 1.01	26.99 ± 0.10	24.17 ± 0.68	30.92 ± 0.07	35.11 ± 3.67	35.76 ± 1.21
ash	1.46 ± 0.16	1.03 ± 0.06	1.19 ± 0.10	1.36 ± 0.07	1.14 ± 0.30	1.07 ± 0
**Gonad**						
moisture	55.63 ± 1.52	53.68 ± 1.71	55.02 ± 0.53	73.50 ± 0.31	72.76 ± 0.35	73.34 ± 0.36
crude protein	28.84 ± 0.08 ^b^	29.86 ± 0.25 ^a^	29.31 ± 0.11 ^ab^	17.61 ± 0.01 ^b^	18.01 ± 0.02 ^a^	17.63 ± 0.05 ^b^
crude lipid	7.45 ± 0.09	6.72 ± 0.32	7.22 ± 0.11	0.34 ± 0	0.42 ± 0.04	0.35 ± 0
ash	2.17 ± 0.40	1.90 ± 0.05	2.23 ± 0.34	2.46 ± 0.32	2.33 ± 0.04	2.25 ± 0.01
**Muscle**						
moisture	79.61 ± 0.25 ^b^	80.88 ± 0.59 ^ab^	81.01 ± 0.44 ^a^	79.33 ± 0.64	78.04 ± 0.50	78.96 ± 0.50
crude protein	17.42 ± 0.05 ^a^	15.71 ± 0.15 ^b^	15.63 ± 0.30 ^b^	16.50 ± 0.06 ^c^	17.98 ± 0.00 ^a^	17.05 ± 0.11 ^b^
crude lipid	0.38 ± 0.03	0.46 ± 0	0.37 ± 0.03	0.35 ± 0.02 ^b^	0.48 ± 0 ^a^	0.38 ± 0.02 ^b^
ash	1.51 ± 0.02	1.50 ± 0.03	1.49 ± 0.07	1.35 ± 0.05	1.50 ± 0.01	1.47 ± 0.02

Notes: The data presented as means ± standard error (SE) (n = 20). Values in the same row with different superscripts are significantly different (*p* < 0.05). Forty mature individuals were collected from each culture environment. Abbreviations: FW, freshwater; SW, saline water; AW, alkaline water.

**Table 2:** The fatty acid composition in hepatopancreas, gonad, and muscle of adult *Eriocheir sinensis* fattening in different culture environments (% of total fatty acids).

Fatty Acid	Hepatopancreas			Gonad			Muscle		
	FW	SW	AW	FW	SW	AW	FW	SW	AW
**Female**									
C14:0	1.36 ± 0.12	1.40 ± 0.04	1.62 ± 0.04	0.85 ± 0.02	0.83 ± 0.01	1.01 ± 0.18	—	—	—
C15:0	0.67 ± 0.06	0.68 ± 0.02	0.76 ± 0.01	0.50 ± 0.02	0.53 ± 0.01	0.55 ± 0.08	—	—	—
C16:0	19.68 ± 0.75	19.46 ± 0.22	20.85 ± 1.74	16.03 ± 0.37	15.50 ± 0.07	16.20 ± 0.40	12.53 ± 0.25	12.37 ± 0.32	13.22 ± 0.25
C17:0	0.74 ± 0.03 ^a^	0.62 ± 0 ^b^	0.71 ± 0.03 ^ab^	0.76 ± 0.05	0.63 ± 0.02	0.74 ± 0.11	0.95 ± 0.02 ^ab^	0.86 ± 0 ^b^	1.01 ± 0.04 ^a^
C18:0	3.07 ± 0.02	3.19 ± 0.07	3.42 ± 0.19	3.15 ± 0.17	2.82 ± 0.02	2.87 ± 0.12	9.79 ± 0.02 ^ab^	9.21 ± 0.21 ^b^	10.08 ± 0.19 ^a^
∑SFA	26.54 ± 0.88	26.45 ± 0.04	28.52 ± 1.89	21.38 ± 0.63	20.47 ± 0.08	21.45 ± 0.88	24.01 ± 0.24 ^ab^	23.12 ± 0.50 ^b^	25.11 ± 0.15 ^a^
C15:1n5	0.30 ± 0.03	0.28 ± 0.02	0.32 ± 0.02	—	—	—	0.61 ± 0.03	0.66 ± 0.01	0.79 ± 0.07
C16:1n7	8.70 ± 0.82	9.57 ± 0.92	9.76 ± 1.32	7.80 ± 0.95	10.33 ± 0.13	10.27 ± 0.48	2.97 ± 0.19	2.81 ± 0.05	2.86 ± 0.46
C18:1n9c	38.59 ± 0.27	36.55 ± 1.04	37.94 ± 1.21	34.18 ± 0.31 ^a^	31.40 ± 0.75 ^b^	32.55 ± 0.46 ^ab^	25.27 ± 0.32	24.92 ± 0.51	25.45 ± 0.14
C20:1n9	1.33 ± 0.08	1.04 ± 0.04	1.18 ± 0.14	0.48 ± 0.05 ^a^	0.33 ± 0.02 ^b^	0.40 ± 0.01 ^ab^	0.76 ± 0.03	0.75 ± 0.02	0.74 ± 0.03
∑MUFA	49.89 ± 0.71	48.34 ± 0.15	50.22 ± 2.72	42.96 ± 1.22	42.57 ± 0.60	43.72 ± 0.05	29.86 ± 0.54	29.46 ± 0.60	30.16 ± 0.24
C18:2n6c	17.06 ± 1.91	19.26 ± 0.47	15.97 ± 4.03	16.96 ± 0.08	17.83 ± 0.26	16.84 ± 1.41	11.25 ± 0.90	10.60 ± 0.26	9.69 ± 0.04
C18:3n3	1.84 ± 0.03	2.12 ± 0.20	1.59 ± 0.36	3.10 ± 0.11	3.85 ± 0.23	3.31 ± 0.18	1.58 ± 0.10	1.61 ± 0.01	1.36 ± 0.18
C20:2n6	1.18 ± 0.03 ^a^	0.90 ± 0.02 ^b^	0.87 ± 0.06 ^b^	1.07 ± 0.07	0.92 ± 0.04	0.90 ± 0.01	1.68 ± 0.05	1.62 ± 0.03	1.58 ± 0.14
C20:4n6 (ARA)	0.96 ± 0.09	0.80 ± 0.10	0.76 ± 0.09	3.38 ± 0.03	3.32 ± 0.11	3.29 ± 0.09	6.73 ± 0.17	7.02 ± 0.18	7.14 ± 0.24
C20:3n3	0.37 ± 0 ^a^	0.30 ± 0.03 ^b^	0.26 ± 0.01 ^b^	0.49 ± 0.04	0.47 ± 0	0.45 ± 0.02	0.56 ± 0.02 ^b^	0.61 ± 0.01 ^a^	0.58 ± 0.01 ^ab^
C20:5n3 (EPA)	1.03 ± 0.04	0.84 ± 0.14	0.72 ± 0.18	6.48 ± 0.06	6.58 ± 0.22	6.35 ± 0.07	16.50 ± 0.28	17.15 ± 0.16	16.34 ± 0.33
C22:6n3 (DHA)	0.44 ± 0.12	0.41 ± 0.08	0.36 ± 0.07	3.24 ± 0.18	3.36 ± 0.18	2.81 ± 0.09	7.62 ± 0.48	8.22 ± 0.22	7.68 ± 0.21
∑PUFA	23.60 ± 0.72	25.21 ± 0.05	21.15 ± 2.06	35.35 ± 0.28	36.86 ± 0.23	34.51 ± 0.42	46.32 ± 0.25 ^ab^	47.23 ± 0.04 ^a^	44.83 ± 0.13 ^b^
∑EFA	19.18 ± 0.84	21.58 ± 0.12	17.78 ± 1.95	20.29 ± 0.08	21.86 ± 0.22	20.34 ± 0.55	13.04 ± 0.44	12.40 ± 0.12	11.31 ± 0.09
∑HUFA	5.09 ± 0.16	4.86 ± 0.29	4.09 ± 0.34	17.08 ± 0.24	17.93 ± 0.15	16.57 ± 0.23	33.19 ± 0.14 ^b^	34.82 ± 0.06 ^a^	33.30 ± 0.09 ^b^
∑n-3 PUFA	3.67 ± 0.13	3.66 ± 0.32	2.93 ± 0.43	13.30 ± 0.27	14.26 ± 0.13	12.91 ± 0.24	26.25 ± 0.08 ^b^	27.58 ± 0.05 ^a^	25.95 ± 0.03 ^b^
∑n-6 PUFA	19.93 ± 1.04	21.55 ± 0.19	18.22 ± 2.31	22.05 ± 0.14	22.60 ± 0.20	21.60 ± 0.74	20.80 ± 0.40	19.65 ± 0.02	18.89 ± 0.19
n-3/n-6 PUFA	0.19 ± 0.03	0.17 ± 0.02	0.16 ± 0	0.60 ± 0.01	0.63 ± 0	0.60 ± 0.05	1.31 ± 0.05	1.40 ± 0	1.37 ± 0.03
∑DHA + EPA	1.46 ± 0.15	1.25 ± 0.22	1.08 ± 0.25	9.72 ± 0.23 ^ab^	9.94 ± 0.04 ^a^	9.16 ± 0.16 ^b^	24.11 ± 0.20 ^b^	25.37 ± 0.05 ^a^	24.02 ± 0.13 ^b^
DHA/EPA	0.42 ± 0.10	0.48 ± 0.02	0.51 ± 0.03	0.50 ± 0.02	0.51 ± 0.04	0.44 ± 0.01	0.46 ± 0.04	0.48 ± 0.02	0.47 ± 0.02
**Male**									
C14:0	1.56 ± 0.07	1.37 ± 0.13	1.62 ± 0.11	0.52 ± 0.07	0.41 ± 0.05	0.49 ± 0.01	—	—	—
C15:0	0.83 ± 0.04 ^a^	0.64 ± 0.01 ^b^	0.73 ± 0.05 ^ab^	—	—	—	—	—	—
C16:0	19.92 ± 0.83 ^a^	17.15 ± 0.07 ^b^	17.78 ± 0.24 ^ab^	10.25 ± 0.39	9.63 ± 0.44	10.21 ± 0.02	13.31 ± 0.45	12.18 ± 0.04	12.60 ± 0.05
C17:0	0.91 ± 0.03 ^a^	0.69 ± 0.01 ^b^	0.87 ± 0.06 ^a^	0.90 ± 0.05	0.75 ± 0.05	0.84 ± 0.01	1.10 ± 0.02	0.93 ± 0.04	1.11 ± 0.09
C18:0	3.63 ± 0.06 ^a^	3.13 ± 0.12 ^b^	3.40 ± 0.02 ^ab^	7.79 ± 0.50	7.48 ± 0.04	7.62 ± 0.07	10.27 ± 0.09	9.49 ± 0.07	10.21 ± 0.30
∑SFA	27.90 ± 0.90 ^a^	23.96 ± 0 ^b^	25.42 ± 0.42 ^ab^	19.90 ± 0.09	18.80 ± 0.58	19.63 ± 0.11	25.33 ± 0.48 ^a^	23.07 ± 0.12 ^b^	24.50 ± 0.35 ^ab^
C15:1n5	0.39 ± 0.02 ^a^	0.28 ± 0.02 ^b^	0.32 ± 0.02 ^ab^	0.78 ± 0.04	0.83 ± 0.02	0.80 ± 0.03	0.92 ± 0.02	0.83 ± 0.03	0.93 ± 0.10
C16:1n7	8.56 ± 0.29	8.04 ± 1.14	9.40 ± 0.82	2.49 ± 0.39	2.38 ± 0.13	3.00 ± 0.13	2.87 ± 0.44	2.36 ± 0.09	2.29 ± 0.11
C18:1n9c	34.99 ± 1.21	32.33 ± 1.28	31.71 ± 0.44	24.23 ± 0.38 ^a^	22.65 ± 0.36 ^b^	24.39 ± 0.17 ^a^	25.43 ± 0.21 ^a^	23.41 ± 0.04 ^c^	24.43 ± 0.12 ^b^
C20:1n9	1.43 ± 0.05 ^a^	1.07 ± 0.11 ^b^	0.97 ± 0.02 ^b^	1.22 ± 0.03	1.09 ± 0.06	1.13 ± 0.01	0.79 ± 0.04	0.75 ± 0.01	0.76 ± 0.02
∑MUFA	46.65 ± 1.61	42.76 ± 0.24	43.37 ± 0.46	29.21 ± 0.77	27.39 ± 0.49	29.80 ± 0.32	30.47 ± 0.77 ^a^	27.72 ± 0.09 ^b^	28.79 ± 0.12 ^ab^
C18:2n6c	16.18 ± 2.20	22.52 ± 0.38	19.53 ± 1.37	8.74 ± 1.14	10.71 ± 0.17	9.83 ± 0.18	10.11 ± 0.58 ^b^	12.18 ± 0.33 ^a^	9.90 ± 0.20 ^b^
C18:3n3	2.56 ± 0.30	3.17 ± 0.44	3.45 ± 0.22	1.13 ± 0.22	1.07 ± 0.04	1.16 ± 0.16	1.60 ± 0.16	1.56 ± 0.09	1.37 ± 0.06
C20:2n6	1.01 ± 0.05	0.95 ± 0.18	0.83 ± 0.01	2.54 ± 0.13	2.63 ± 0.11	2.55 ± 0.18	1.50 ± 0.08 ^b^	1.72 ± 0.03 ^a^	1.64 ± 0.02 ^ab^
C20:4n6 (ARA)	1.61 ± 0	1.65 ± 0.05	1.92 ± 0.13	15.21 ± 0.89	13.97 ± 0.40	14.54 ± 0.53	7.63 ± 0.21	7.36 ± 0.23	8.03 ± 0.26
C20:3n3	0.37 ± 0.01	0.38 ± 0.02	0.38 ± 0.01	0.66 ± 0.01 ^a^	0.55 ± 0.01 ^b^	0.63 ± 0.02 ^a^	0.61 ± 0.03	0.59 ± 0.01	0.62 ± 0.02
C20:5n3 (EPA)	1.36 ± 0.09	1.85 ± 0.21	2.09 ± 0.16	10.22 ± 0.97	10.79 ± 0.20	10.40 ± 0.30	13.87 ± 0.19 ^b^	15.48 ± 0.20 ^a^	15.68 ± 0.26 ^a^
C22:6n3 (DHA)	1.40 ± 0.01 ^b^	1.96 ± 0.14 ^a^	2.17 ± 0.08 ^a^	6.53 ± 0.38	6.82 ± 0.32	6.00 ± 0.07	8.64 ± 0.17	9.77 ± 0.26	9.35 ± 0.31
∑PUFA	25.45 ± 1.13 ^b^	33.32 ± 0.10 ^a^	31.23 ± 0.40 ^ab^	45.37 ± 0.47	46.81 ± 0.36	45.49 ± 0.14	44.20 ± 0.13 ^c^	48.84 ± 0.10 ^a^	46.82 ± 0.06 ^b^
∑EFA	19.00 ± 1.10	25.90 ± 0.02	23.19 ± 0.51	10.22 ± 0.60	12.05 ± 0.10	11.39 ± 0	11.96 ± 0.17 ^b^	13.93 ± 0.19 ^a^	11.51 ± 0.14 ^b^
∑HUFA	8.01 ± 0.20 ^b^	9.65 ± 0.40 ^ab^	10.67 ± 0.24 ^a^	33.75 ± 1.01	33.20 ± 0.44	32.72 ± 0.01	32.34 ± 0.16 ^b^	34.75 ± 0.30 ^a^	35.04 ± 0.07 ^a^
∑n-3 PUFA	5.69 ± 0.28 ^b^	7.35 ± 0.54 ^ab^	8.08 ± 0.21 ^a^	18.54 ± 0.80	19.23 ± 0.34	18.19 ± 0.39	24.71 ± 0.08 ^b^	27.39 ± 0.25 ^a^	27.01 ± 0.09 ^a^
∑n-6 PUFA	19.76 ± 1.23	25.97 ± 0.32	23.15 ± 0.68	26.84 ± 0.05	27.58 ± 0.19	27.31 ± 0.49	19.49 ± 0.23 ^b^	21.45 ± 0.08 ^a^	19.81 ± 0 ^b^
n-3/n-6 PUFA	0.29 ± 0.01	0.28 ± 0.04	0.35 ± 0.03	0.69 ± 0.04	0.70 ± 0.01	0.67 ± 0.04	1.27 ± 0.03	1.28 ± 0.03	1.36 ± 0.01
∑DHA + EPA	2.76 ± 0.10 ^b^	3.80 ± 0.35 ^a^	4.26 ± 0.08 ^a^	16.75 ± 1.35	17.61 ± 0.52	16.40 ± 0.36	22.51 ± 0.02 ^b^	25.25 ± 0.46 ^a^	25.03 ± 0.05 ^a^
DHA/EPA	1.03 ± 0.06	1.07 ± 0.05	1.05 ± 0.12	0.64 ± 0.02	0.63 ± 0.02	0.58 ± 0.01	0.62 ± 0.02	0.63 ± 0.01	0.60 ± 0.03

Notes: The data presented as means ± standard error (SE) (n = 20). Fatty acids less than 0.3% were not listed in the table or instead of —. Values in the same row with different superscripts are significantly different (*p* < 0.05). Thirty mature individuals were collected from each culture environment. Abbreviations: FW, freshwater; SW, saline water; AW, alkaline water; ∑SFA, sum of saturated fatty acids; ∑MUFA, sum of monounsaturated fatty acids; ∑PUFA, sum of polyunsaturated fatty acids; ∑EFA, sum of essential fatty acids; ∑HUFA, sum of highly unsaturated fatty acids; ∑n-3 PUFA, sum of ω-3 polyunsaturated fatty acids; ∑n-6 PUFA, sum of ω-6 polyunsaturated fatty acids.

**Table 3:** Free amino acid composition in hepatopancreas, gonad, and muscle of adult *Eriocheir sinensis* fattening in different culture environments (mg/100 g, wet weight).

Free Amino Acids	Hepatopancreas			Gonad			Muscle		
	FW	SW	AW	FW	SW	AW	FW	SW	AW
**Female**									
Aspartic acid	41.33 ± 3.44 ^b^	58.10 ± 2.80 ^a^	61.89 ± 3.31 ^a^	10.23 ± 2.87	7.04 ± 1.28	8.21 ± 2.04	3.72 ± 0.58	2.98 ± 0.26	3.59 ± 0.22
Arginine	299.51 ± 28.50	275.89 ± 1.46	286.47 ± 13.84	223.54 ± 18.42	291.75 ± 29.03	227.79 ± 18.41	487.49 ± 1.84 ^b^	452.77 ± 17.15 ^b^	544.70 ± 10.59 ^a^
Alanine	164.85 ± 11.12 ^a^	121.78 ± 7.78 ^b^	135.10 ± 4.74 ^ab^	75.95 ± 12.48	82.20 ± 24.79	66.25 ± 2.35	303.43 ± 24.60	317.08 ± 18.38	354.05 ± 32.29
Cysteine	11.55 ± 0.19 ^b^	16.21 ± 0.75 ^a^	15.54 ± 0.20 ^a^	0.97 ± 0.65	0.41 ± 0.16	1.02 ± 0.08	3.14 ± 0.96	3.30 ± 0.46	3.41 ± 0.93
Glutamic acid	104.54 ± 8.34	111.19 ± 4.96	115.50 ± 3.16	104.86 ± 8.72	129.77 ± 34.62	120.67 ± 12.59	38.13 ± 8.16	51.94 ± 3.41	61.20 ± 17.91
Glycine	80.13 ± 0.10	80.09 ± 9.62	81.19 ± 3.07	43.89 ± 9.91	51.84 ± 14.00	43.84 ± 3.18	450.43 ± 33.41 ^b^	595.76 ± 7.73 ^a^	461.48 ± 32.33 ^b^
Histidine	32.51 ± 4.82 ^b^	49.74 ± 3.11 ^a^	47.94 ± 2.46 ^ab^	25.62 ± 1.86 ^b^	33.12 ± 0.52 ^a^	27.95 ± 0.57 ^ab^	16.56 ± 0.72	22.12 ± 1.52	22.44 ± 2.56
Proline	96.27 ± 14.42 ^b^	203.41 ± 17.57 ^a^	202.96 ± 8.17 ^a^	131.94 ± 17.98 ^b^	272.57 ± 32.06 ^ab^	315.82 ± 47.81 ^a^	238.99 ± 40.97 ^b^	367.13 ± 51.10 ^ab^	491.15 ± 44.92 ^a^
Serine	18.09 ± 2.18	13.58 ± 0.90	14.40 ± 0.37	8.48 ± 2.01	6.86 ± 0.65	4.92 ± 0.01	10.08 ± 1.21	9.51 ± 1.25	6.68 ± 0.15
Tyrosine	76.31 ± 1.43 ^b^	86.43 ± 2.69 ^a^	92.36 ± 0.44 ^a^	20.80 ± 8.10	18.64 ± 2.57	13.30 ± 0.86	15.11 ± 2.14	15.69 ± 2.24	21.44 ± 3.70
Isoleucine ^▲^	39.21 ± 2.26 ^b^	75.79 ± 1.00 ^a^	49.94 ± 3.33 ^b^	10.49 ± 2.94	10.34 ± 0.90	7.87 ± 0.19	10.86 ± 1.24	13.99 ± 2.29	18.23 ± 2.74
Leucine ^▲^	112.38 ± 6.02 ^b^	167.73 ± 0.95 ^a^	151.50 ± 1.42 ^a^	21.59 ± 9.25	22.55 ± 0.58	15.32 ± 1.56	23.33 ± 2.59	32.57 ± 6.10	40.77 ± 5.96
Lysine ^▲^	158.23 ± 6.43 ^b^	164.07 ± 4.15 ^ab^	183.05 ± 0.78 ^a^	51.43 ± 6.59 ^b^	93.31 ± 6.33 ^a^	71.28 ± 1.37 ^ab^	33.16 ± 2.43 ^b^	39.17 ± 5.33 ^ab^	72.64 ± 11.85 ^a^
Methionine ^▲^	41.07 ± 1.63 ^ab^	45.69 ± 2.36 ^a^	34.73 ± 2.61 ^b^	16.94 ± 5.20	10.69 ± 1.67	9.11 ± 0.02	21.84 ± 0.31	19.75 ± 1.11	27.15 ± 3.86
Phenylalanine ^▲^	71.82 ± 1.68 ^b^	94.11 ± 0.05 ^a^	94.11 ± 0.41 ^a^	19.83 ± 6.62	19.57 ± 1.26	11.83 ± 0.98	13.40 ± 2.08	15.13 ± 2.95	20.63 ± 3.27
Threonine ^▲^	61.61 ± 1.55 ^b^	79.43 ± 1.42 ^a^	83.23 ± 4.56 ^a^	56.60 ± 0.62	79.45 ± 11.39	59.13 ± 6.06	23.66 ± 1.08	31.92 ± 7.65	38.70 ± 7.42
Valine ^▲^	67.23 ± 1.89	69.82 ± 0.68	71.94 ± 4.20	26.69 ± 4.85	21.57 ± 0.62	15.48 ± 1.56	30.23 ± 1.87	38.04 ± 5.00	39.45 ± 7.44
∑EFAA	551.55 ± 18.37 ^b^	696.63 ± 5.77 ^a^	668.50 ± 12.09 ^a^	203.56 ± 34.83	257.49 ± 22.75	190.03 ± 11.74	156.47 ± 11.60	190.56 ± 30.43	257.58 ± 42.54
∑FAA	1476.65 ± 4.09 ^b^	1713.04 ± 10.57 ^a^	1721.86 ± 46.53 ^a^	849.86 ± 80.54	1151.68 ± 95.42	1019.80 ± 94.39	1723.54 ± 40.57 ^b^	2028.83 ± 84.18 ^a^	2227.72 ± 12.45 ^a^
PETFAA	37.35 ± 1.14	40.67 ± 0.59	38.83 ± 0.35	23.78 ± 1.85 ^a^	22.35 ± 0.12 ^ab^	18.69 ± 0.58 ^b^	9.07 ± 0.46	9.35 ± 1.11	11.55 ± 1.84
**Male**									
Aspartic acid	45.09 ± 1.29	53.68 ± 7.18	55.49 ± 5.88	49.91 ± 3.14	61.92 ± 5.04	66.65 ± 2.83	2.29 ± 0.36	3.20 ± 0.51	2.98 ± 0.63
Arginine	255.53 ± 29.47	247.79 ± 22.87	223.99 ± 6.52	47.87 ± 1.16	45.28 ± 2.40	51.45 ± 2.66	489.02 ± 32.00	537.25 ± 16.27	490.85 ± 65.55
Alanine	141.21 ± 7.13	108.50 ± 11.38	126.99 ± 6.63	71.09 ± 7.57	59.15 ± 3.12	78.11 ± 3.14	300.88 ± 20.95	314.91 ± 49.62	308.30 ± 36.41
Cysteine	13.94 ± 0.18	15.88 ± 1.73	16.23 ± 0.67	3.03 ± 0.09 ^a^	0.12 ± 0.01 ^c^	2.15 ± 0.16 ^b^	0.95 ± 0.48	2.84 ± 0.65	2.49 ± 0.24
Glutamic acid	88.52 ± 13.75	98.54 ± 1522	99.91 ± 14.26	58.79 ± 7.22	50.39 ± 1.58	61.69 ± 3.12	54.32 ± 9.05	74.84 ± 10.81	69.55 ± 9.58
Glycine	66.90 ± 0.94	78.89 ± 0.58	77.14 ± 8.51	27.57 ± 2.43	26.67 ± 0.91	27.77 ± 0.30	437.69 ± 28.26	561.13 ± 33.12	546.93 ± 33.55
Histidine	35.56 ± 5.40	44.41 ± 4.66	45.11 ± 4.08	8.75 ± 0.61 ^c^	11.42 ± 0.16 ^b^	13.72 ± 0.17 ^a^	13.17 ± 1.84 ^b^	21.18 ± 1.86 ^a^	15.31 ± 0.77 ^ab^
Proline	109.18 ± 0.87	253.22 ± 19.02	262.20 ± 83.68	73.92 ± 12.06	108.29 ± 13.53	137.26 ± 35.29	208.61 ± 21.22 ^b^	473.18 ± 77.05 ^a^	476.86 ± 51.06 ^a^
Serine	16.03 ± 1.43	13.70 ± 1.22	18.50 ± 0.16	3.25 ± 0.03	3.51 ± 0.57	4.10 ± 0.62	8.19 ± 1.47	8.31 ± 0.10	5.51 ± 1.25
Tyrosine	86.85 ± 2.53	80.89 ± 8.96	80.48 ± 5.16	19.99 ± 1.37	19.55 ± 0.41	19.56 ± 0.22	13.24 ± 1.10 ^b^	21.22 ± 0.65 ^a^	17.37 ± 2.43 ^ab^
Isoleucine ^▲^	44.24 ± 5.23	50.56 ± 5.39	54.31 ± 5.51	10.55 ± 0.68 ^b^	12.49 ± 0.37 ^b^	15.74 ± 0.82 ^a^	9.60 ± 2.17	13.45 ± 1.08	12.32 ± 2.79
Leucine ^▲^	126.43 ± 2.35	157.29 ± 10.77	140.48 ± 11.37	15.40 ± 2.35 ^b^	17.07 ± 0.53 ^ab^	21.96 ± 0.62 ^a^	22.63 ± 3.96	28.93 ± 1.39	26.94 ± 5.53
Lysine ^▲^	174.21 ± 9.13	143.66 ± 16.03	141.24 ± 7.96	26.24 ± 2.88	24.41 ± 0.97	27.66 ± 0.97	44.22 ± 2.36	48.05 ± 2.59	55.60 ± 6.77
Methionine ^▲^	41.06 ± 0.05	43.75 ± 6.20	38.58 ± 2.94	5.87 ± 0.90	4.70 ± 0.70	5.86 ± 0.14	14.99 ± 0.35	22.58 ± 4.11	17.47 ± 0.19
Phenylalanine ^▲^	82.88 ± 2.13	88.34 ± 10.24	86.42 ± 8.34	8.15 ± 0.79	8.93 ± 0.46	10.51 ± 0.10	10.21 ± 1.64	15.42 ± 0.46	12.63 ± 1.75
Threonine ^▲^	70.37 ± 12.98	73.90 ± 8.24	80.67 ± 4.43	9.53 ± 0.15 ^c^	13.38 ± 0.27 ^b^	16.66 ± 0.98 ^a^	21.79 ± 4.77	30.70 ± 0.71	35.17 ± 5.18
Valine ^▲^	67.41 ± 3.90	70.02 ± 6.31	72.26 ± 4.05	14.90 ± 0.07 ^a^	9.92 ± 0.82 ^b^	11.86 ± 0.37 ^b^	29.14 ± 6.17	38.67 ± 5.90	34.42 ± 6.19
∑EFAA	606.61 ± 35.67	627.53 ± 18.24	613.95 ± 44.59	90.65 ± 7.52	90.89 ± 3.38	110.27 ± 3.99	152.58 ± 21.42	197.80 ± 9.65	194.54 ± 28.02
∑FAA	1465.42 ± 54.30	1623.03 ± 111.06	1620.00 ± 180.13	454.82 ± 23.95	477.20 ± 10.18	572.73 ± 50.49	1680.96 ± 73.19	2215.86 ± 200.09	2130.69 ± 229.01
PETFAA	41.36 ± 0.90	38.77 ± 1.53	38.06 ± 1.48	20.07 ± 2.71	19.04 ± 0.30	19.34 ± 1.01	9.04 ± 0.88	8.96 ± 0.37	9.09 ± 0.34

Notes: The data presented as means ± standard error (SE) (n = 20). ^▲^, Essential amino acid. Values in the same row with different superscripts are significantly different (*p* < 0.05). Thirty mature individuals were collected from each culture environment. Abbreviations: FW, freshwater; SW, saline water; AW, alkaline water; ∑EFAA, total essential free amino acids; ∑FAA, total free amino acids; PETFAA, percentage of ∑EFAA to ∑FAA.

**Table 4:** The threshold and taste activity value of free amino acid composition in hepatopancreas, gonad, and muscle of adult *Eriocheir sinensis* fattening in different culture environments.

Free Amino Acids	Flavor Characteristics	Threshold (mg/100 mL)	Hepatopancreas			Gonad			Muscle		
			FW	SW	AW	FW	SW	AW	FW	SW	AW
**Female**											
Aspartic acid	umami (+)	100	0.41	0.58	0.62	0.10	0.07	0.08	0.04	0.03	0.04
Glutamic acid	umami (+)	30	3.48	3.71	3.85	3.50	4.33	4.02	1.27	1.73	2.04
∑TUV			3.89	4.29	4.47	3.60	4.40	4.10	1.31	1.76	2.08
Alanine	sweetness (+)	60	2.75	2.03	2.25	1.27	1.37	1.10	5.06	5.28	5.90
Glycine	sweetness (+)	130	0.62	0.62	0.62	0.34	0.40	0.34	3.46	4.58	3.55
Serine	sweetness (+)	150	0.12	0.09	0.10	0.06	0.05	0.03	0.07	0.06	0.04
Threonine	sweetness (+)	260	0.24	0.31	0.32	0.22	0.31	0.23	0.09	0.12	0.15
Proline	sweetness/bitterness (+)	300	0.32	0.68	0.68	0.44	0.91	1.05	0.80	1.22	1.64
∑TSV			4.05	3.73	3.97	2.33	3.04	2.75	9.48	11.26	11.28
Arginine	sweetness/bitterness (−)	50	5.99	5.52	5.73	4.47	5.83	4.56	9.75	9.06	10.89
Lysine	sweetness/bitterness (−)	50	3.16	3.28	3.66	1.03	1.87	1.43	0.66	0.78	1.45
Valine	sweetness/bitterness (−)	40	1.68	1.75	1.80	0.67	0.54	0.39	0.76	0.95	0.99
Methionine	bitterness/sweetness/sulphur (−)	30	1.37	1.52	1.16	0.56	0.36	0.30	0.73	0.66	0.91
Histidine	bitterness (−)	20	1.63	2.49	2.40	1.28	1.66	1.40	0.15	0.17	0.23
Isoleucine	bitterness (−)	90	0.44	0.84	0.55	0.12	0.11	0.09	0.12	0.16	0.20
Leucine	bitterness (−)	190	0.59	0.88	0.80	0.11	0.12	0.08	0.12	0.17	0.21
Phenylalanine	bitterness (−)	90	0.80	1.05	1.05	0.22	0.22	0.13	0.83	1.11	1.12
∑TBV			15.66	17.33	17.15	8.46	10.71	8.38	13.12	13.06	16.00
**Male**											
Aspartic acid	umami (+)	100	0.45	0.54	0.55	0.50	0.62	0.67	0.02	0.03	0.03
Glutamic acid	umami (+)	30	2.95	3.28	3.33	1.96	1.68	2.06	1.81	2.49	2.32
∑TUV			3.40	3.82	3.88	2.46	2.30	2.73	1.83	2.52	2.35
Alanine	sweetness (+)	60	2.35	1.81	2.12	1.18	0.99	1.30	5.01	5.25	5.14
Glycine	sweetness (+)	130	0.51	0.61	0.59	0.21	0.21	0.21	3.37	4.32	4.21
Serine	sweetness (+)	150	0.11	0.09	0.12	0.02	0.02	0.03	0.05	0.06	0.04
Threonine	sweetness (+)	260	0.27	0.28	0.31	0.04	0.05	0.06	0.08	0.12	0.14
Proline	sweetness/bitterness (+)	300	0.36	0.84	0.87	0.25	0.36	0.46	0.70	1.58	1.59
∑TSV			3.60	3.63	4.01	1.70	1.63	2.06	9.21	11.33	11.12
Arginine	sweetness/bitterness (−)	50	5.11	4.96	4.48	0.96	0.91	1.03	9.78	10.75	9.82
Lysine	sweetness/bitterness (−)	50	3.48	2.87	2.82	0.52	0.49	0.55	0.88	0.96	1.11
Valine	sweetness/bitterness (−)	40	1.69	1.75	1.81	0.37	0.25	0.30	0.73	0.97	0.86
Methionine	bitterness/sweetness/sulphur (−)	30	1.37	1.46	1.29	0.20	0.16	0.20	0.50	0.75	0.58
Histidine	bitterness (−)	20	1.78	2.22	2.26	0.44	0.57	0.69	0.11	0.17	0.14
Isoleucine	bitterness (−)	90	0.49	0.56	0.60	0.12	0.14	0.17	0.11	0.15	0.14
Leucine	bitterness (−)	190	0.67	0.83	0.74	0.08	0.09	0.12	0.12	0.15	0.14
Phenylalanine	bitterness (−)	90	0.92	0.98	0.96	0.09	0.10	0.12	0.66	1.06	0.77
∑TBV			15.51	15.63	14.96	2.78	2.71	3.18	12.89	14.96	13.56

Notes: (+). pleasant taste; (−). unpleasant taste. ND, taste threshold not detected. Thirty mature individuals were collected from each culture environment. Abbreviations: FW, freshwater; SW, saline water; AW, alkaline water; ∑TUV, total umami values; ∑TSV, total sweetness values; ∑TBV, total bitterness values.

## Data Availability

The data presented in this study are available on request from the corresponding author.
